# Mechanical Properties, Cytotoxicity, and Fluoride Ion Release Capacity of Bioactive Glass-Modified Methacrylate Resin Used in Three-Dimensional Printing Technology

**DOI:** 10.3390/ma15031133

**Published:** 2022-02-01

**Authors:** Zbigniew Raszewski, Julita Kulbacka, Agnieszka Nowakowska-Toporowska

**Affiliations:** 1 SpofaDental, Markova 238, 506-01 Jicin, Czech Republic; Zbigniew.Raszewski@kavokerr.com; 2 Department of Molecular and Cellular Biology, Faculty of Pharmacy, Wroclaw Medical University, Borowska 211A, 50-556 Wroclaw, Poland; julita.kulbacka@umed.wroc.pl; 3 Department of Prosthodontics, Wroclaw Medical University, 50-425 Wroclaw, Poland

**Keywords:** 3D printing, Poly(methyl methacrylate) resin (PMMA), bioactive material, mechanical properties, cytotoxicity

## Abstract

Background: Clinically, three-dimensional (3D) printing technology is becoming a popular and efficient dental processing technology. Recently, there has been an increasing demand for dental materials that exhibit bioactive properties. The present study aimed to evaluate the mechanical properties, cytotoxicity, and fluoride ion release capacity of 3D-printed dental resins modified with bioactive glass. Materials and methods: The resin FotoDent splint used in the production of removable orthodontic splints, was modified by the addition of two types of bioactive glasses that are capable of releasing fluoride ions. The novel materials used for the production of dental splints were examined for their mechanical, physical, and biological properties (fracture resistance, sorption, solubility, elution of nonpolymeric substances, and release of fluoride ions over time) and cytotoxic effects on cell cultures. Results: Initially, the fracture toughness of the 3D-printed resin was found to be 55 MPa, but after modification with glass, the resistance was reduced to about 50 MPa. Sorption and solubility values of the materials (19.01 ÷ 21.23 µg/mm^3^ and 0.42 ÷ 1.12 µg/mm^3^, respectively) complied with the safety limits imposed by ISO standard. Modified resins were capable of releasing fluoride ions, and the maximum releasing effect was observed after 14 days of incubation. Both the modified resins, after four days of contact with human gingival fibroblasts, exhibited moderate cytotoxic properties. Conclusions: The experimental results showed that modification of methacrylate resin, used in 3D printing technology, with bioactive glasses produces novel dental materials that possess desirable bioactive properties. The findings of this study indicate the potential ability of modified polymethacrylate resins to release fluoride ions in the oral cavity environment. The modified materials are characterized with a moderate decrease in physical properties and mild cytotoxicity on direct contact with human fibroblasts.

## 1. Introduction

Currently, three-dimensional (3D) printing technology is extensively used in the field of dentistry. Although various techniques and printers are available and have been used for the production of light-cured PMMA resin and sintering metallic powders, attempts have also been made to print ceramics [1,2]. Presently, the most widely used technique is stereolithographic printing of light-cured resins [3,4,5]. Although several technologies are used to print dental composites in 3D printers, the basic composition of the resin material remains the same in all the methods. PMMA resins used for 3D printing technology are composed of chemical compounds that are specifically used for printing diagnostic models, surgical templates, mouth guards for contact sports, and orthodontic appliances [3]. Photocurable materials used for printing denture plates/crowns and bridges are also available on the dental market [6,7,8,9].

The mechanical properties of these materials have been extensively studied and described in the literature. The dental materials used for the preparation of resins should possess desirable characteristics such as adequate flexural strength (FS), hardness, impact strength, and color stability. In addition, the photocured resins usually exhibit various cytotoxic effects, which is a challenging problem. Piironen et al. [10], Kumar [11], and Ahamed [12] evaluated biological properties, such as cytotoxicity and elution of different unpolymerized components, of resins used for 3D printers. Recently, there has been rapid development in the synthesis of novel bioactive materials that have a wide range of applications. The most important examples of bioactive materials are glass ionomers and composite resins modified with hydroxyapatite, bioactive glasses, and other compounds [13,14]. These biocompatible products are capable of releasing various types of ions, such as calcium, phosphorus, and fluoride, into the oral cavity. These ions are known to inhibit teeth demineralization and initiate teeth remineralization and restoration process. Furthermore, the fluoride ions act as effective anticaries agents. Various dental materials have been modified to prolong the fluoride-releasing action of resins. Some of the modification techniques involve the addition of sodium fluoride or other fluoride salts and incorporation of nanoparticles of calcium fluoride, full prereacted glass-ionomer fillers (F-PRG), and surface PRG cements (S-PRG) [15].

The results of previous studies have shown that the development of novel dental materials from light-polymerized resins enriched with bioactive properties may be feasible by using advanced 3D printing technology [16]. Such bioactive materials would be beneficial for the patients who use removable partial dentures, orthodontic and prosthetic splints, or mouth guards. Their presence in the oral cavity disrupts the natural saliva flow to the places where the resin appliances come in direct contact with the natural teeth. The lack of saliva flow to these areas leads to a decrease in the pH value over the period of time, which can subsequently cause the growth of the microbes responsible for caries formation [17]. The main compounds used in the synthesis of 3D-printed resins are acrylate and methacrylate resins, and it is a well-known fact that these materials do not show bacteriostatic properties when used for a prolonged period of time [5,9].

The present study aimed to create novel dental materials for orthodontic splint production used in 3D printing technology enriched with novel bioactive glass fillers of different composition. The aim was also to assess their physical properties, cytotoxicity effects, and fluoride ions’ release capacity. Various parameters, such as fracture resistance, sorption, solubility, capacity to release fluoride ions, elution of nonpolymerized components, and their influence on cell cultures, were evaluated for both unmodified and modified resin products. The null hypothesis assumed in this study was that there were no differences in the physical properties, fluoride ion release ability, and cytotoxic properties between the modified and unmodified materials.

## 2. Materials and Methods

FotoDent splint (Dreve, Unna, Germany) is a light-cured methacrylate resin that is used for the production of removable orthodontic splints, and this dental device was selected for conducting further tests in this study. In the first stage, the test resin material was divided into three portions. Two portions were modified with the addition of 10 wt% Kavitan and 10 wt% Fritex bioactive glass powders (SpofaDental, Jicin, Czech Republic), while one portion was not modified with any compound (control group). The composition of bioactive glasses is presented in Table 1.

The particle size of glass medium was 8.56 µm for Kavitan and 6.5 µm for Fritex (tested by CILAS 1064 granulometer; Pittsburg, PA, USA). The composites used for investigation purposes were prepared by mixing 90 g of FotoDent splint resin with 10 g of glass powder in plastic containers made of black polyethylene. To facilitate uniform dispersion of the components, the mixture was supplemented with five Al_2_O_3_ balls (Jezierska Porcelanka, Desná, Czech Republic) having a diameter of 5 mm. Then, the ingredients were mixed on a roller mixer machine (Jezierska Porcelanka, Desná, Czech Republic) at 40 rpm for 60 min [18]. The prepared suspensions were transferred into the resin containers of the printer Liquid Crystal Precision (Photocentric Ltd., Peterborough, England), which uses Daylight Polymer Printing technology for curing photoreactive polymers. The irradiation time for printing of single layer of 25 µm thickness was 2 s, and the wavelength was 372 nm. The specimens were designed with 3D Builder (10.1.9.0, Microsoft Corporation, Redmont, WA, USA. The specimens printed from unmodified resin constituted the reference group.

**Figure 1:**
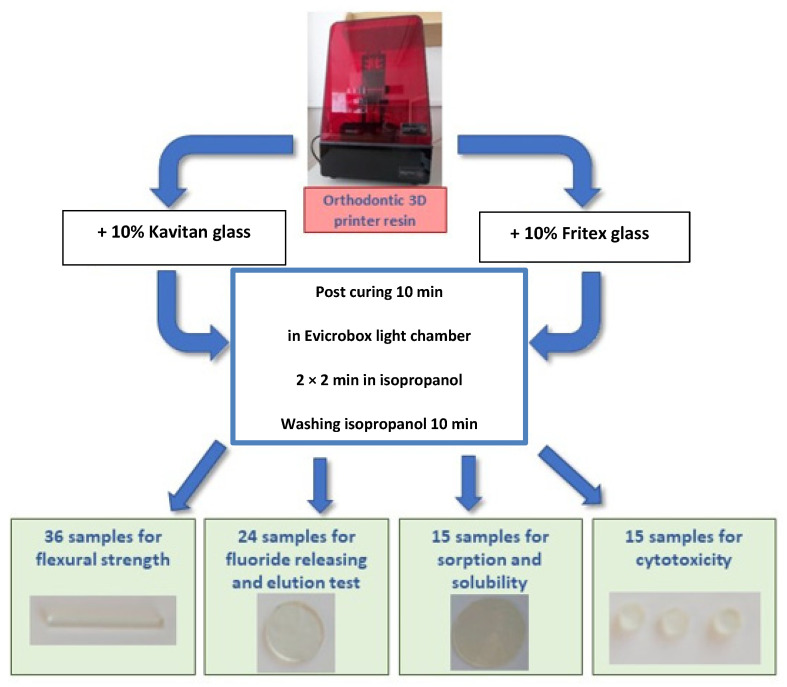
The scheme of specimen preparation for conducting various experiments in this study.

A total of 90 samples, which were obtained from the three types of tested materials, (unmodified resin, Kavitan modified resin, and Fritex modified resin), were designed using computer software and printed using a printer. Thirty-six rectangular samples with dimensions of 20 ± 0.1 × 2 ± 0.1×25 ± 0.1 mm^3^ were produced to assess their ability to resist fracture. Twenty-four cylinder-shaped samples were printed with a diameter of 6 ± 0.1 mm and a thickness of 1 ± 0.1 mm to investigate the leaching of nonpolymerized components and fluoride ion releasing ability. Fifteen cylinder-shaped specimens were printed with a diameter of 50 ± 0.1 mm and a thickness of 1 ± 0.1 mm to test the sorption and solubility properties. Fifteen cylinder-shaped specimens with a diameter of 4 ± 0.1 mm and a thickness of 2 ± 0.1 mm were produced for cytotoxicity studies. To remove the remaining unpolymerized resins, samples were transferred to the isopropanol solution in an ultrasonic cleaner and washed two times for 2 min, as recommended by the manufacturer. After the initial hardening process in a 3D printer (Liquid Crystal Precision, Photocentric Ltd, Peterborough, England), the specimens were irradiated for 10 min in a light chamber Evicrobox (SpofaDental, Jicin, Czech Republic) with the light source emitting light in the range of 350–450 nm. After completing the curing process, the support structures were removed. The steps involved in the preparation of dental specimens are presented in Figure 1.

### 2.1. Fracture Resistance

Fracture resistance test was performed at two time intervals, that is, after 24 h of storage in distilled water (six specimens were included in each group), and the second test was conducted after 30 days (six specimens were included in each group). Shimadzu compressive strength instrument (AGS 10 kNG, Shimadzu, Kyoto, Japan) was used to determine the fracture resistance. The fracture strength of dental resins was evaluated by performing a 3-point bending test. The tensile strength was tested by maintaining the loading rate at 0.5 mm/min. The distance between the supports was adjusted to 20 mm. The fracture resistance was calculated by determining the dimensions of the sample according to the formula proposed by EN ISO 4049:2019 standard guidelines [19].

$$\mathrm{FS}=\frac{3\times 20\times F}{2h\times d\times d}$$

where *FS* is the flexural strength in MPa, *F* is the force at breaking point in *N*, *h* is the thickness in mm, and *d* is the width in mm.

### 2.2. Sorption and Solubility

Sorption and solubility tests were performed in accordance with the EN ISO 20795-1:2013 [20] standard guidelines. Initially, cylindrical specimens with the required diameter were weighed daily on an analytical balance (Precioza 256; Turin, Italy) until a constant weight was achieved (M1). Later, the samples were placed in containers containing distilled water for 7 days at 37 °C and then weighed again immediately after removing them from the water (M2). In the next stage, the discs were dried in a desiccator and weighed daily until a constant mass was established (M3). Sorption and solubility properties were calculated using the formula proposed by the EN ISO 20795-1:2013 standard guidelines.

$$\mathrm{Sorption}\;[µg/{\mathrm{mm}}^{3}]=\frac{M2-M1}{\mathrm{specimen}\;\mathrm{volume}}\;\mathrm{Solubility}\;[µg/{\mathrm{mm}}^{3}]=\frac{M1-M3}{\mathrm{specimen}\;\mathrm{volume}}$$

### 2.3. Leaching of Nonpolymerized Components

A spectrophotometric method was used to analyze the elution of the nonpolymerized compounds according to the protocol proposed by González et al. [21]. The leaching levels of nonpolymerized components was determined for each sample (three samples were included for each group of the tested material) by incubating the material in 10 mL of an extraction solvent containing 99% ethanol solution (Lacher; Prague, Czech Republic) for 2 h. Later, 5 mL of the eluted solution was taken, and the absorbance was measured at a wavelength range of 190–1100 nm (ultraviolet-visible range) using a Helios spectrophotometer (Thermo Fisher Scientific, Waltham, MA, USA). A 5 mL quartz cuvette was used for the measurements. Pure ethanol was taken in a second cuvette and was used as a reference solvent.

### 2.4. Determination of Fluoride Ion Release

The spectrophotometric dye method was used to determine the amount of the fluoride ions released from the tested materials according to the method described by Unal et al. [22]. A standard calibration curve was prepared using solutions containing different known concentrations of fluoride ions (1, 2, 10, 20, and 50 ppm). The absorbance values were recorded at a wavelength of 524 nm, as the color complex showed maximal absorbance at this wavelength. Helios spectrophotometer (Thermo Fisher Scientific, Waltham, MA, USA) was used to measure the spectral absorption.

Modified and unmodified resin (used as reference material) materials were placed in separate containers containing 10 mL of distilled water. After incubation for 24 h, 7 and 14 days, water was removed from the specimens and used for elution tests. To 1 mL of eluent, 2 mL of 0.01 M iron chloride solution (Sigma Aldrich, Prague, Czech Republic) and 1 mL of 0.01 M 3-indoleacetic acid (Sigma Aldrich, Prague, Czech Republic) were added in an acidic environment. The whole mixture was heated at 60 °C for 10 min until a pink color complex was formed. Subsequently, 1 mL of distilled water was added to the pink-colored solution to obtain a final volume of 5 mL. The intensity of the pink color complex was inversely proportional to the concentration of fluoride ions in the mixture.

### 2.5. Cell Culture

Human gingival fibroblasts (HGFs) were used to conduct the experiments in this study. This primary cell culture was isolated from a fragment of gingival tissues, and the procedure was described previously by Saczko et al. [23]. The cells were grown in 25 cm^2^ flasks (EQUIMED, Sarstedt, Germany) containing Dulbecco’s Modified Eagle’s Medium (DMEM; IITD, Wroclaw, Poland) supplemented with 10% FBS (Sigma, Burlington, MA, USA) penicillin/streptomycin solution (Sigma, Burlington, MA, USA), and 1% GlutaMAXTM-I (Gibco, El Paso, TX, USA). The cells were maintained in a humidified atmosphere at 37 °C and 5% CO_2_. For experimental procedures, cells were detached from the surface by trypsinization (0.25% Trypsin/EDTA; IITD, Wroclaw, Poland).

### 2.6. Evaluation of Cell Morphology and Migration

The impact of the investigated resin materials on the target cells was evaluated. The cells were cultivated in standard Dulbecco’s Modified Eagle’s Medium (DMEM; Sigma, Burlington, MA, USA) in the presence of tested materials. For the documentation of cell growth, the tested materials were plated on a 24-well plate (Biocom-Nunc, Janki, Poland). Then HGF cells were seeded at a density of 1 × 10^4^ cells/well in a culture medium. The culture plates were incubated in a humidified atmosphere at 37 °C and 5% CO_2_. The microscopic observations were performed after 1 and 24 h of exposure to the tested materials (Leica DMi1; CellService, Poznan, Poland).

### 2.7. Cytotoxicity Assay Test—Direct Contact

For the cytotoxicity studies, three specimens with a diameter of 5 mm and a thickness of 2 mm were prepared. The impact of the tested resin materials on the growth of gingival fibroblasts was evaluated after 24 h of exposure using PrestoBlue^®^ assay or after 96 h of exposure using methyl thiazolyl diphenyl tetrazolium bromide (MTT; Sigma Aldrich, Burlington, MA, USA) assay. Initially, the investigated resin samples were plated on a 24-well plate (Biocom-Nunc, Janki, Poland). Then HGF cells were seeded at a density of 1 × 10^4^ cells/well in a culture medium. After 24 h of incubation, the culture medium was removed and subsequently analyzed by PrestoBlue^®^ assay. PrestoBlue^®^ (ThermoFisher, Warsow, Poland) is a ready-to-use cell-permeable resazurin-based solution that functions as a cell viability indicator by using the reducing power of living cells to measure the proliferation of cells quantitatively. The culture medium was incubated with the reagent for 1 h. The fluorescent signal was recorded on a multiplate reader (GloMax^®^ Discover; Promega, Madison, MI, USA) using an excitation wavelength of 520 nm and an emission filter of 580–640 nm. The experiment was performed in triplicate for each of the tested resin samples.

After incubating the fibroblast cells in the presence of resin samples for 96 h, the MTT assay was performed using 2-isopropanol in the final step (Alchem, Torun, Poland). Then, the sample solutions were collected from the wells, and their absorbance was measured at 560 nm on a microplate reader (GloMax^®^ Discover; Promega, Madison, WI, USA). The results obtained from spectrophotometric and spectrofluorimetric measurements were used to determine the number of viable cells by comparing the experimental values with the values obtained for control untreated cells. Mean values were used for further calculations.

### 2.8. Statistical Analysis

The results were statistically analyzed using one-way analysis of variance at a significance level of 0.05. In addition, a post hoc analysis was performed by using Tukey’s HSD test (using a free test calculator provided by Astatsa, San Jose, CA, USA).

## 3. Results

The results of FS tests are presented in Table 2. Statistically significant differences were observed between the unmodified 3D resin (control group) and resin modified with 10 wt% Kavitan glass and 10 wt% Fritex glass.

A decrease of about 5 MPa was noticed in the FS of the tested material after the addition of bioactive glass powders. The commercially available orthodontic resins have an FS of more than 50 MPa. After the specimens were stored in water for 30 days at 37 °C, the fracture resistance of all the materials was slightly reduced. This finding can be attributed to the absorption of water by the materials with increasing time of storage in distilled water.

**Figure 2:**
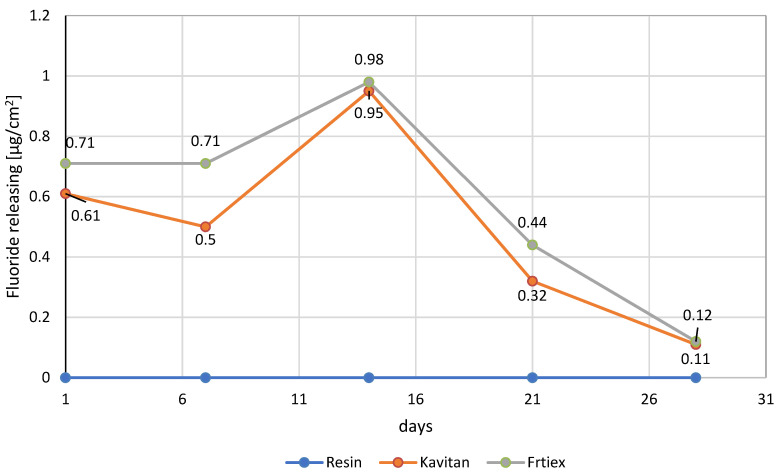
The evaluation of fluoride ions released from the resins over time.

Resins that were modified with bioactive glasses showed higher sorption and solubility when compared to the properties exhibited by unmodified resin. This observation can be mainly attributed to the migration of ions from the glass particles from the resin material. The group including resin materials modified with Kavitan glass showed higher values for these parameters (solubility: 1.12 ± 0.13 µg/mm^3^, sorption: 21.23 ± 3.57 µg/mm^3^) when compared to the values revealed by the other two groups. Statistically significant differences were observed for materials modified with bioactive glass fillers when compared to unmodified resin. However, no differences were found between the modified groups (Table 3). All the values of sorption and solubility of all tested materials confirmed with ISO EN ISO 4049:2019 requirements and were below 32 µg/mm^3^ for sorption and 1.6 µg/mm^3^ for solubility. Solubility is specifically related to the process of release of fluoride ions by the dental material, and the results are shown in Figure 2.

When pure resin samples were washed with isopropanol solution and measured in a spectrophotometer, peaks corresponding to catalyst and methacrylate (acrylates)-containing ring structures were observed in this study (Table 4). The product safety data indicates that the peaks observed at wavelengths 243 and 282 nm represent (5-ethyl-1,3-dioxan-5-yl)methyl acrylate and 2-phenoxyethyl methacrylate, respectively. The compound diphenyl (2,4,6-trimethylbenzoyl) phosphine oxide contributes to the absorption at a wavelength of 372 nm. Leaching of nonpolymerized components also affects the sorption and solubility properties of the tested dental materials after seven days, and the results are presented in Table 3.

The unmodified resin samples did not release any fluoride ions, while the modified resin samples were able to emit fluoride ions. A maximum value was obtained for both the tested materials after 14 days of incubation with both glass powders (Resin + 10 wt% Kavitan: 0.95 ± 0.06 µg/cm^2^, Resin + 10 wt% Fritex: 0.98 ± 0.13 µg/cm^2^), and subsequently a gradual decrease in the amount of released fluoride ions was noticed.

**Figure 3:**
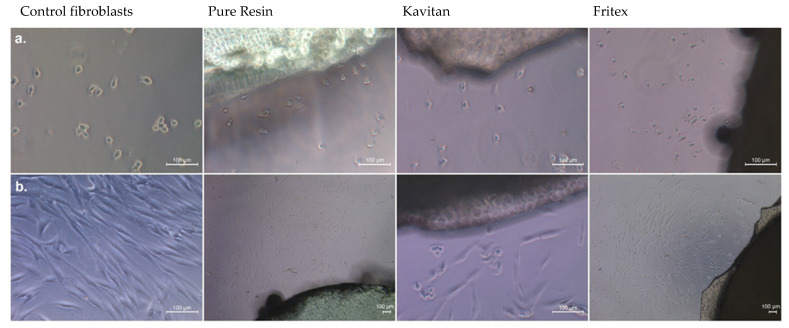
The biocompatibility assay of the primary culture of HGFs after 1 and 2 h of incubation (magnification 200 ×; Leica).

The evaluation results of cell morphology and migration are presented in Figure 3. It is evident that after exposure for a shorter period of time (1 h, upper panel in Figure 3), the cells still appeared in round shape but started to attach to the surface of the culture dish. When the exposure time was doubled, a significant increase in the adhesion of cells to the bottom of the plate was observed, and an elevation in the cell number was also noticed. At this stage, cells appeared elongated and regular, with normal cell division process and no inhibition of cell growth.

**Figure 4:**
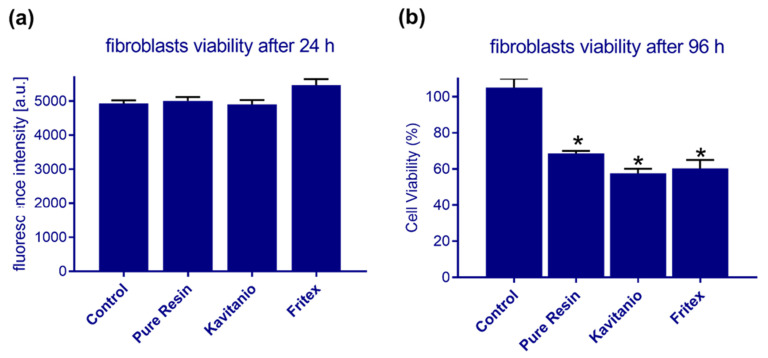
The results of the viability assay of fibroblasts after exposure to the tested materials for (**a**) 24 h (by PrestoBlue assay) and (**b**) 96 h (by MTT assay).(*) indicates statistically significant differences among tested groups.

Figure 4 presents the cytotoxicity effects of modified resins on cell cultures, the statistically significant differences among groups was indicated with (*) symbol. The growth of the cells was not affected after 24 h of incubation, but a significant inhibition was observed after 96 h when compared to the effects produced by the reference material.

## 4. Discussion

Currently, different types of materials that are commonly used for manufacturing dental devices and instruments possess bioactive properties. They are known to release various ions into the oral cavity, which provide significant benefit for the patient because these ions can help in the prevention of enamel demineralization and caries development [24,25].

The null hypothesis proposed at the beginning of this study was rejected because significant differences were observed in the mechanical properties, fluoride ion release capacity, and cytotoxicity effects between the modified and unmodified 3D orthodontic splint resins.

Various bioactive materials have been used for the production of orthodontic splints, which are devices used for the treatment of certain types of dental problems. The treatment requires that the splints be replaced every 7–14 days with a better fitting one that exerts more pressure to align the teeth in the desired position [25]. Maximum release of fluoride ions from the modified materials was observed on the 14th day and ranged between 0.95 ± 0.05 and 0.98 ± 0.13 ppm. The previously reported concentration of released fluoride ions is adequate to initiate the healing effect. Margolis et al. suggested that enamel remineralization occurs even at low fluoride ion concentrations (ranging from 0.024 to 0.154 ppm). Some studies reported that a continuous supply of low levels of fluoride ions (ranging from 0.03 to 0.3 ppm) for a prolonged period of time provides more beneficial effects and effectively prevents caries development [24,25].

In this study, the modified resin materials showed fracture resistance that ranged from 50 MPa (after 1 day of storage in distilled water) to 46 MPa (after 30 days of storage). This is acceptable for the manufacture of removable orthodontic devices, where the materials are expected to have a fracture resistance of about 50 MPa. An increase in mechanical resistance can be observed by using a higher concentration of silanized fillers in the 3D resin. However, it may lead to an increase in the viscosity of the resin, which would further affect the efficiency of the 3D printing process [5,26]. Parreira-Lovo et al. examined the mechanical properties of resins that can be potentially used for 3D printing technology. The fracture resistance of various products ranged from 50 to 80 MPa [26].

The dental materials must show good biological compatibility and safety when used in living organisms. The orthodontic resins used in the 3D printing process exhibit mild cytotoxic properties, which was confirmed in this study. Washing of the material with isopropanol facilitated the removal of only unpolymerized monomers and catalysts present superficially on the surface. Therefore, no inhibition in the growth of cell cultures was observed after incubation for a short period of time (2–24 h). However, after 4 days, leaching of nonpolymerized monomers into the solution was noted, which contributed to peak formation in the absorption spectra. This elution process showed a negative impact on the growth of fibroblast cells, which resulted in a 60%–70% reduction in cell viability. This effect can be attributed to the fact that the resins used in 3D printing technology belong to the same chemical group as those used in composite materials and bonding systems. Several studies have been carried out previously to determine the effect of unpolymerized monomers on the cytotoxic properties following direct contact with the cell cultures [24,25,26,27,28,29,30]. With the passage of time and after the completion of polymerization process, more compounds may be eluted into the oral cavity, which are eventually washed away by saliva. Therefore, the biocompatibility of materials composed of methacrylate monomers increases with time [29]. Kumar, in his study, compared the cytotoxicity of three types of materials used for the manufacturing of orthodontic splints [11]. The tested materials were Invisalign^®^ (polyurethane-based), Dental LT^®^ resin (Form labs Inc., Somerville, MA, USA), photopolymeric explicit material prepared from methacrylate monomers, and polycarbonate Accura 60^®^ SLA (3D systems, Rock Hill, SC, USA). The author used the MTT staining technique to identify the living cells (3T3 mice fibroblast cells). After 24 h of production of the orthodontic trays from all the tested materials, their cytotoxic effects on the cell cultures were examined. The results showed that the materials based on polyurethanes and acrylic resins showed a survival rate of up to 80% after 7 days. For polycarbonate-based materials, the cytotoxic properties persisted for a longer period of time. The author suggests that this observation can be attributed to the continuous and gradual release of bisphenol A, which is the main component of this type of polymer. Puskar et al. [31] assessed the cytotoxic effects of Accura, which was also determined by a previous study, on two cell cultures: mouse fibroblast (L929) cells and human lung fibroblast (MRC-5) cells. The cell viability of the two cultures was evaluated by performing Mosmann’s colorimetric MTT assay and the agar diffusion test. The tested material showed no cytotoxic properties when incubated for a short time period (1–3 days), but it demonstrated mild cytotoxic effect after 5, 7, and 21 days of incubation for both cell lines (cell viability > 77%). Piironen et al. recommended that the implant materials printed by 3D technique and used as scaffolds for cell growth should be subjected to autoclave sterilization. The efficiency of this method was tested during the production of Dental SG resin [10]. However, all the resins cannot be cured by this process because it can lead to the deformation of resin components [32]. Lin et al. exposed L929 cells to bis EMA, TEGDMA, and UDMA methacrylic resins that are used in the 3D printers. The authors proved that these materials are biocompatible and that the cell culture survival rate was over 70% by performing MTT assay, although the monomer conversion rate was approximately 50%–60%. This may be due to the presence of unpolymerized high molecular weight compounds in the solution even after alcohol bath and their occurrence in the spaces between the polymer chains in the cube surfaces, which are small enough to prevent the leaching of monomers into the surrounding environment [32]. The results obtained in this study with regard to the cytotoxicity effects of the tested materials are consistent with the observations of other authors. A fundamental issue associated with the biocompatibility of resins is that the materials should be properly cured in light chambers after their removal from the 3D printers [27,33]. The second crucial step is washing the finished products in alcohol solutions to remove unpolymerized monomers and catalysts. However, their presence in the tested materials was confirmed in this study, as well as in studies conducted by other authors [6,10,11]. Washing with the alcohol solution also facilitated the removal of nonpolymeric oxygen inhibition layer from the surface of printed objects, which significantly increased the cytotoxicity effects of the material [29]. Another parameter that may affect the biocompatibility of materials is the type of catalyst used and its concentration. The lower the concentration of the catalyst, the longer the exposure time that is required to obtain a higher degree of conversion of the methacrylate bonds [21]. The methacrylate monomers used in the preparation of 3D printer resins significantly influence the cytotoxicity behavior and the mechanical properties of the dental materials.

## 5. Conclusions

The experimental results obtained from this study showed that modification of methacrylate resin, used in 3D printing technology, with two bioactive glass fillers produces novel dental materials that possess desirable bioactive properties. The findings of this study indicate the potential ability of modified polymethacrylate resins to release fluoride ions in the oral cavity environment. The reported concentration of released fluoride ions is adequate to initiate the enamel remineralization process. The modified materials are characterized with a moderate decrease of flexural strength. Sorption and solubility levels of the modified materials were higher than of the nonmodified material but still within the accepted limits. Leaching of nonpolymerized components was also noted. All of the tested materials had mild cytotoxicity on direct contact with human fibroblasts.

## Figures and Tables

**Table 1:** Composition of bioactive glasses [wt%].

Kavitan	SiO_2_ (26 ± 3%)	Al_2_O_3_ (29 ± 3%)	F (10%)	P_2_O_5_ (8%)	Na_2_O (4%)	SrO (17%)
Fritex	SiO_2_ (44 ± 3%)	Al_2_O_3_ (30 ± 3%)	F (6%)	P_2_O_5_ (3%)	Na_2_O (6%)	CaO (5%)

**Table 2:** Flexural strength of tested materials after 1 day and 30 days.

Composition	Flexural Strength [MPa] after 1 day	Flexural Strength [MPa] after 30 Days
3D Resin	54.99 ± 2.72 Aa	46.29 ± 3.69 Ab
3D Resin + 10% Fritex glass	50.54 ± 2.97 Ba	43.69 ± 2.87 Bb
3D Resin + 10% Kavitan glass	49.68 ± 3.61 Ba	43.92 ± 2.10 Bb

Note: Small letters in rows and big letters in columns denote statistically significant differences at *p* ≤ 0.05.

**Table 3:** Solubility and sorption properties.

Composition	Solubility [µg/mm^3^] after 7 Days	Sorption [µg/mm^3^] after 7 Days
3D Resin	0.42 ± 0.09 A	15.76 ± 4.21 A
3D Resin + 10% Fritex glass	1.03 ± 0.11 B	19.01 ± 2.14 B
3D Resin + 10% Kavitan glass	1.12 ± 0.13 B	21.23 ± 3.57 B

Note: Big letters in columns denote statistically significant differences at *p* ≤ 0.05.

**Table 4:** Results obtained after the ethanol extraction of unpolymerized compounds from the tested resins.

Unmodified 3D Resin	3D Resin + 10% Fritex	3D Resin + 10% Kavitan
Wave length [nm] Absorption [A] 243 0.061 ± 0.012 282 0.146 ± 0.014 372 0.102 ± 0.016	Wave length [nm] Absorption [A] 243 0.046 ± 0.017 282 0.104 ± 0.013 382 0.129 ± 0.011	Wave length [nm] Absorption [A] 243 0.074 ± 0.013 282 0.164 ± 0.016 382 0.168 ± 0.013
